# Analysis of sex differences in dietary copper-fructose interaction-induced alterations of gut microbial activity in relation to hepatic steatosis

**DOI:** 10.1186/s13293-020-00346-z

**Published:** 2021-01-06

**Authors:** Ming Song, Fang Yuan, Xiaohong Li, Xipeng Ma, Xinmin Yin, Eric C. Rouchka, Xiang Zhang, Zhongbin Deng, Russell A. Prough, Craig J. McClain

**Affiliations:** 1 Department of Medicine, Division of Gastroenterology, Hepatology and Nutrition, University of Louisville School of Medicine, Louisville, KY 40202 USA; 2 Hepatobiology&Toxicology Program, University of Louisville, Louisville, KY 40202 USA; 3 University of Louisville Alcohol Research Center, University of Louisville, Louisville, KY 40202 USA; 4 Department of Chemistry, University of Louisville, Louisville, KY 40208 USA; 5 Center for Regulatory and Environmental Analytical Metabolomics, University of Louisville, Louisville, KY 40208 USA; 6 KBRIN Bioinformatics Core, Louisville, KY 40292 USA; 7 Department of Pharmacology and Toxicology, University of Louisville School of Medicine, Louisville, KY 40202 USA; 8 Department of Microbiology & Immunology, Brown Cancer Center, University of Louisville, Louisville, KY 40202 USA; 9 James Graham Brown Cancer Center, University of Louisville, Louisville, KY 40202 USA; 10 Department of Biochemistry and Molecular Genetics, University of Louisville School of Medicine, Louisville, KY 40202 USA; 11 Robley Rex Veterans Affairs Medical Center, Louisville, KY 40206 USA

**Keywords:** Copper, Fructose, Gut microbiota, Sex, Nonalcoholic fatty liver disease

## Abstract

**Background:**

Inadequate copper intake and increased fructose consumption represent two important nutritional problems in the USA. Dietary copper-fructose interactions alter gut microbial activity and contribute to the development of nonalcoholic fatty liver disease (NAFLD). The aim of this study is to determine whether dietary copper-fructose interactions alter gut microbial activity in a sex-differential manner and whether sex differences in gut microbial activity are associated with sex differences in hepatic steatosis.

**Methods:**

Male and female weanling Sprague-Dawley (SD) rats were fed ad libitum with an AIN-93G purified rodent diet with defined copper content for 8 weeks. The copper content is 6 mg/kg and 1.5 mg/kg in adequate copper diet (CuA) and marginal copper diet (CuM), respectively. Animals had free access to either deionized water or deionized water containing 10% fructose (F) (w/v) as the only drink during the experiment. Body weight, calorie intake, plasma alanine aminotransferase, aspartate aminotransferase, and liver histology as well as liver triglyceride were evaluated. Fecal microbial contents were analyzed by 16S ribosomal RNA (16S rRNA) sequencing. Fecal and cecal short-chain fatty acids (SCFAs) were determined by gas chromatography-mass spectrometry (GC-MS).

**Results:**

Male and female rats exhibit similar trends of changes in the body weight gain and calorie intake in response to dietary copper and fructose, with a generally higher level in male rats. Several female rats in the CuAF group developed mild steatosis, while no obvious steatosis was observed in male rats fed with CuAF or CuMF diets. Fecal 16S rRNA sequencing analysis revealed distinct alterations of the gut microbiome in male and female rats. Linear discriminant analysis (LDA) effect size (LEfSe) identified sex-specific abundant taxa in different groups. Further, total SCFAs, as well as, butyrate were decreased in a more pronounced manner in female CuMF rats than in male rats. Of note, the decreased SCFAs are concomitant with the reduced SCFA producers, but not correlated to hepatic steatosis.

**Conclusions:**

Our data demonstrated sex differences in the alterations of gut microbial abundance, activities, and hepatic steatosis in response to dietary copper-fructose interaction in rats. The correlation between sex differences in metabolic phenotypes and alterations of gut microbial activities remains elusive.

**Supplementary Information:**

The online version contains supplementary material available at 10.1186/s13293-020-00346-z.

## Introduction

The prevalence of nonalcoholic liver disease (NAFLD) in the USA has increased rapidly in the past two decades, from 19 to 24%, which is close to the global prevalence of 25.24% [1, 2]. Based on the epidemiological data from obesity and type 2 diabetes in adults, the estimated prevalence of NAFLD will continue to increase up to 33.5% by 2030, and nonalcoholic steatohepatitis (NASH) will increase proportionately from 20% of NAFLD to 27%, ranking it as a top indication for liver transplantation [3, 4].

Of note, NAFLD and NASH exhibit age and sex differences, with a higher prevalence in men than in premenopausal women. Conversely, a higher rate of NAFLD was found among the postmenopausal women [5–7]. In agreement with this finding, sex differences also exist in the risk factors, such as obesity and type 2 diabetes [8, 9]. Biological sex differences are exhibited in many physiological phenomenon, including fat distribution, triglyceride storage in the liver and muscle [10], and fatty acid and glucose metabolism [11]. Therefore, understanding sex differences in physiology and pathophysiology is required for precision medicine.

Sex hormones and sex chromosome are two major factors driving sex differences [7]. The role of sex hormones has been demonstrated in both human and animal studies. For example, postmenopausal women with estrogen deficiency display a higher risk for NAFLD progression to fibrosis [12]. In contrast, liver injury was improved by hormone replacement therapy in postmenopausal women with type 2 diabetes [13]. Ovariectomized (OVX) female rats exhibit exacerbated hepatic steatosis when exposed to high-fat high-fructose diet (HFFD), which was reversed by estrogen replacement [14]. A four-core genotype mouse model (XX gonadal male and female, XY gonadal male and female) allows for the identification of whether sex differences arise from the sex chromosome complement. Using this approach, it was revealed that XX mice are prone to developing obesity and fatty liver in response to high-fat diet, regardless of sex hormones [15].

In addition to genetics and sex hormones, diet is a key environmental factor leading to sex differences in metabolic diseases [16]. Copper and fructose are two dietary factors known to be critical in the pathogenesis of NAFLD [17–22]. Sex differences in the metabolic effects of fructose and/or copper deficiency have been noted in rodents [23–26] as well as in humans [27, 28], with more harmful effects reported in males and more protective effects in females, which is consistent with the sex differences in NAFLD [7]. In fact, sex differences in fructose-induced metabolic effects are more complex and vary by tissue and organ [14, 29, 30]. Although sex hormones are one of the factors leading to sex differences in copper-fructose interaction-induced metabolic disorders [26], the underlying mechanisms are largely unknown.

A growing body of evidence has shown that gut microbiota play a causal role in driving the development of obesity, diabetes, and NAFLD [31–34]. Diet, as one of the most common environmental factors, shapes the gut microbiome [35]. Interestingly, diet-induced alterations of gut microbiota exhibit a sex-dependent phenotype [36, 37]. Previous studies have shown that distinct alterations of the gut microbiome are linked to specific metabolic traits [38] as well as to different stages of NAFLD [39, 40], leading to the hypothesis that sex differences in the gut microbiota are linked to distinct metabolic phenotypes or disease severity. Our previous studies have shown that dietary copper-fructose interactions shifted gut microbiota and correlated to the development of hepatic steatosis in male rats [41, 42]. Given that diet shapes the gut microbiome in a sex-specific manner [36], we aimed to determine whether dietary copper-fructose interaction alters gut microbiota and induces hepatic steatosis in a sex-dependent manner and whether sex differences in metabolic phenotype contribute to the distinct alterations of the gut microbiota.

## Materials and methods

### Animals and diets

Male and female weanling Sprague-Dawley rats (35–45 g) from the Harlan Laboratories (Indianapolis, IN) were fed (ad lib) an AIN-93G purified rodent diet with a defined copper content. The rats received either 1.5 mg/kg or 6.0 mg/kg of copper as marginal or adequate doses, respectively, for 8 weeks. Control animals were fed adequate copper with no added fructose. The animals were single housed in stainless steel cages without bedding in a temperature- and humidity-controlled room with a 12:12-h light–dark cycle. Animals had free access to either deionized water or deionized water containing 10% fructose (w/v). Fructose-enriched drinking water was changed twice a week. Food consumption and body weight were monitored on a weekly basis. After a 2-h fasting, all the animals were sacrificed under anesthesia with ketamine/xylazine (100/10 mg/kg I.P. injection). Blood was collected from the inferior vena cava, and citrated plasma was stored at − 80 °C for further analysis. Portions of liver tissue were fixed with 10% formalin for subsequent sectioning, while others were snap-frozen with liquid nitrogen. All studies were approved by the University of Louisville Institutional Animal Care and Use Committee, which is certified by the American Association of Accreditation of Laboratory Animal Care.

### Liver enzyme and plasma biochemical assays

Liver enzymes assays were performed with commercially available kits: alanine aminotransferase (ALT), aspartate aminotransferase (AST), cholesterol, triglyceride (TG) (Thermo Fisher Scientific Inc., Middletown, VA, USA), glucose (Millipore Sigma, St. Louis, MO, USA), and nonesterified fatty acids (NEFA) (Wako Chemicals, Richmond, VA, USA).

### Histology

Formalin-fixed, paraffin-embedded liver sections were cut at 5-μm thickness and stained with hematoxylin and eosin (H&E).

### Hepatic triglyceride assay

Liver tissues were homogenized in 50 mM sodium chloride solution. Hepatic total lipids were extracted with chloroform/methanol (2:1) according to the method described by Bligh and Dyer [43]. Hepatic triglyceride was determined by commercially available kit (Thermo Fisher Scientific Inc., Middletown, VA, USA).

### 16S ribosomal RNA (16S rRNA) gene library preparation and sequencing on the Illumina MiSeq

Fecal pellets were collected into sterile tubes at the end of the experiment and stored at − 80 °C. Microbial genomic DNA was extracted from frozen fecal samples using DNeasy PowerSoil kit (Cat#:12888-100, Qiagen, Germantown, MD, USA) according to the manufacturer’s instructions. The composition of fecal microbiota was analyzed using Illumina MiSeq technology targeting the variable V3 and V4 regions of 16S ribosomal RNA. 16S variable regions were amplified using 12.5 ng microbial genomic DNA. PCR conditions are as follows: 95 °C for 3 min; 25 cycles of 95 °C for 30 s, 55 °C for 30 s, and then 72 °C for 30 s; and 72 °C for 5 min. The primers used for 16S Amplicon PCR are as follows: Forward: 5′-TCGTCGGCAGCGTCAGATGTGTATAAGAGACAGCCTACGGGNGGCWGCAG; Reverse: 5′-GTCTCGTGGGCTCGGAGATGTGTATAAGAGACAGGACTACHVGGGTATCTAATCC. Index PCR was performed to attach dual indices and Illumina sequencing adapters using the Nextera Index Kit (Cat#: FC-121-1012, Illumina, San Diego, CA, USA). Each step was followed by the PCR clean-up, using AMPure XP beads to obtain a purified library. After libraries were normalized, pooled, and denatured, sequencing was done using Illumina MiSeq Reagents kit v3 (600 cycles, read lengths up to 2 × 300 bp) (Cat#: MS-102-3003, Illumina, San Diego, CA, USA) on an Illumina MiSeq instrument.

### Sequencing data analysis

Quality control of raw sequence files was performed using FastQC and further analyzed using QIIME 2 (version 2019.04) [44]. The workflow is shown in the schematic diagram (supplementary Figure 1). Briefly, the paired-end files per sample were merged and imported into a QIIME 2 artifact. The sequences reads were then demultiplexed and denoised into amplicon sequence variants (ASVs) (supplementary Table 8) using DADA2 in QIIME 2 which can identify more real variants and output fewer spurious sequences than other methods. The resulted feature table and representative sequences were used for the downstream analysis. Rarefaction curve using the observed operational taxonomy unit (OTU) and Shannon index generated by QIIME 2 were used as metrics of α-diversity [45]. Principal coordinate analysis (PCoA) was performed to compare microbial community structure between groups (β-diversity), using both weighted and unweighted UniFrac [46]. Heat map analysis of OTU abundance was performed using R software (https://www.r-project.org/). Linear discriminant analysis (LDA) effect size (LEfSe) method was used to find the most differentially abundant enriched microbial taxa between the different diets. The analysis was performed on Galaxy platform (http:/huttenhower.sph.harvard.edu/galaxy). The data generated from LEfSe analysis was shown by cladogram and histogram with LDA score > 2 and a significance of *α* < 0.05, as determined by Wilcoxon rank-sum test [47–49]. The 16S data set was used for metagenome predictions using the software package PICRUSt2 [50]. Predictions were based on Kyoto Encyclopedia of Genes and Genomes (KEGG) database pathways [51], and the output was based on the pathway mapping of the MetaCyc database [52]. A Venn diagram was used to show genus distribution between groups.

### Short-chain fatty acid (SCFA) measurement by gas chromatography-mass spectrometry (GC-MS)

About 50 mg of cecal and fecal stool samples were weighed, and polar metabolites were extracted for GC-MS analysis using established methods as described previously [53].

### Statistical analysis

Data were expressed as mean ± SD (standard deviation) and analyzed using two-way ANOVA to test the factors of copper, fructose, and their interactions (copper × fructose), followed by Tukey’s multiple comparison test. The Kruskal-Wallis test was used for pairwise comparison between treatment groups (α-diversity). Comparison of the mean distance matrix (β-diversity) between two treatment groups using PERMANOVA (a nonparametric method for multivariate analysis of variance) with permutation tests was based on UniFrac distance matrix (999 Monte Carlo permutations). Two-tailed nonparametric Spearman correlation was done with GraphPad Prism. Differences at *p* ≤ 0.05 were considered to be statistical significant.

## Results

### Characterization of dietary copper-fructose interaction on metabolic phenotypes in male and female rats

**Fig. 1:**
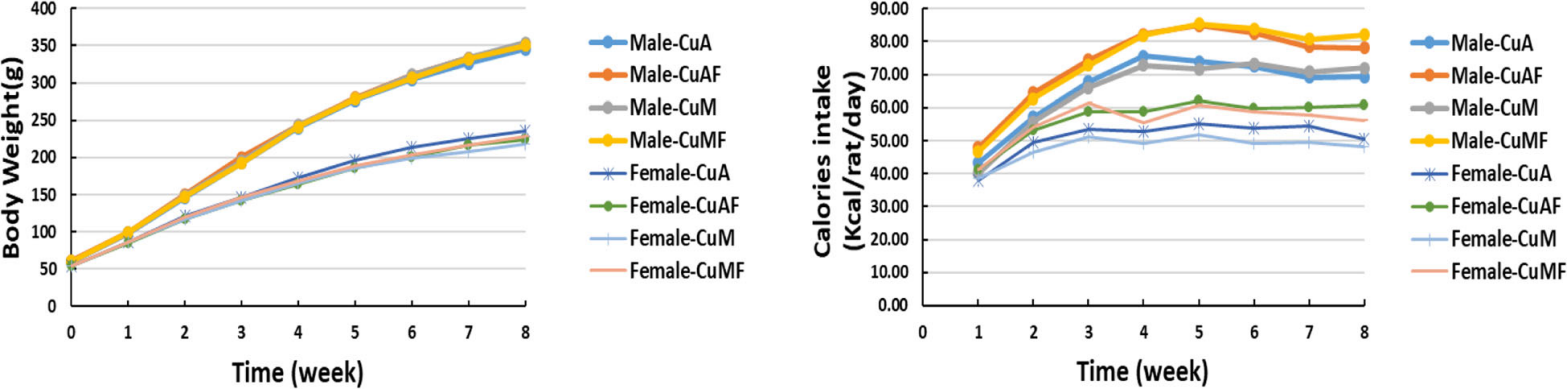
Body weight and calorie intake throughout the 8 weeks of the experiment. Male and female weanling Sprague-Dawley rats were fed with adequate or marginal copper diet and had free access to deionized water or deionized water containing 10% fructose (w/v) for 8 weeks as described in the “Materials and Methods” section. Data represent means ± SD (*n* = 7–8). *Cu*, copper; *A*, adequate copper diet; *AF*, adequate copper diet +10% fructose (w/v) in the drinking water; *M*, marginal copper diet; *MF*, marginal copper diet +10% fructose (w/v) in the drinking water

Male and female rats exhibit similar trends of changes in the body weight and body weight gain in response to dietary copper and fructose, with a generally higher level in male rats (Fig. 1, Tables 1 and 2). Two-way ANOVA analysis showed that the liver weight of female rats, but not male rats, was affected by dietary copper content within the 8-week period. The liver/body weight ratio was altered by both dietary copper and fructose. However, copper-fructose interaction was apparent only in female rats. While the variations of perigonadal white adipose tissue (WAT) weight as well as WAT/body weight ratios were related to dietary copper content in male rats, they were more likely to be affected by dietary fructose in female rats. The energy efficiency ratio (EER, %), i.e., the ratio of body weight gain and total energy intake [54, 55], was decreased by dietary fructose in both male and female rats compared to their controls, suggesting the metabolic effects of fructose may not be contributed to the calorie intake. Ad libitum feeding of fructose via drinking water led to a significant increase in water intake and a decrease in pellet food intake. Although there was a trend toward an increase in the total energy intake in rats fed with fructose compared to those without, the difference did not reach statistical significance in either males or females. Plasma triglyceride was significantly elevated in male rats fed with fructose regardless dietary copper. However, it was only significantly elevated in CuMF female rats compared to marginal copper diet (CuM) female rats. Plasma cholesterol levels were not significantly changed by dietary fructose or copper level in both male and female rats. Plasma NEFA was significantly increased in CuAF male rats compared to adequate copper diet (CuA) rats. In female rats, fructose feeding led to a trend of an increase in plasma NEFA levels. Plasma glucose level was significantly elevated by fructose feeding in female rats regardless of dietary copper level, whereas this effect was only observed in male CuA rats (Tables 1 and 2). Collectively, plasma lipids and glucose display distinct alterations in response to dietary copper and fructose between male and female rats.

**Table 1:** Effects of dietary fructose and marginal copper deficiency on metabolic phenotypes in male rats

Variable	CuA (*n* = 7)	CuAF (*n* = 8)	CuM (*n* = 7)	CuMF (*n* = 8)	*P* value of factors (two-way ANOVA)
Body weight (BW, g)	347 ± 20.6	346.4 ± 19.6	351.7 ± 24.5	346.2 ± 16.8	NS
BW gain (g)	287.9 ± 18.9	285.4 ± 19	291.6 ± 23.1	285.9 ± 18.5	NS
Liver weight (LW, g)	13.07 ± 0.8	13.71 ± 0.79	12.31 ± 1.25	13.15 ± 2.14	NS
LW/BW (%)	3.763 ± 0.115	3.958 ± 0.131	3.501 ± 0.241^#^	3.783 ± 0.438	Cu, *p* = 0.0357 F, *p* = 0.0228
White adipose weight (WAT, g)	3.949 ± 0.383	4.149 ± 0.897	3.347 ± 0.25	3.831 ± 0.529	Cu, *p* = 0.0408
WAT/BW (%)	1.14 ± 0.13	1.19 ± 0.199	0.953 ± 0.048	1.107 ± 0.152	Cu, *p* = 0.0176
Energy efficiency ratio (EER, %)	7.78 ± 0.51	6.87 ± 046*	7.98 ± 0.63^#^	6.85 ± 0.44*^$^	F, *p* < 0.0001
Cecum weight (g)	2.736 ± 0.366	2.528 ± 0.276	2.909 ± 0.294	2.718 ± 0.202	NS
Food consumption (g/rat/day)	17.58 ± 2.88	13.93 ± 2.04*	17.36 ± 3.13	14.06 ± 2.07*	F, *p* = 0.0007
Water intake (ml/rat/day)	26.18 ± 6.31	53.63 ± 17.01*	24.52 ± 5.86^#^	53.73 ± 20.79*^$^	F, *p* < 0.0001
Energy intake (Kcal/rat/day)	66.11 ± 10.81	74.13 ± 12.30	65.26 ± 12.77	74.52 ± 13.50	NS
Plasma TG (mg/dL)	49.01 ± 13.26	95.08 ± 53.56*	36.83 ± 10.66^#^	91.86 ± 25.76^$^	F, *p* = 0.0002
Plasma cholesterol (mg/dL)	53.28 ± 20.19	53.43 ± 23.13	52.58 ± 13.94	41.73 ± 24.4	NS
Plasma NEFA (μM)	203.6 ± 49.3	400.5 ± 144.7*	263.1 ± 97.4	273.8 ± 64.6	F, *p* = 0.0073 Cu x F, *p* = 0.0149
Plasma glucose (mg/dL)	104.9 ± 18.8	147.3 ± 23.7*	119 ± 14.2	129.9 ± 16.4^#^	F, *p* = 0.0012 Cu x F, *p* = 0.0394

Male and female weanling Sprague-Dawley rats from the Harlan Laboratories (Indianapolis, IN) were fed (ad lib) a modified AIN-93G purified rodent diet with defined copper content in the form of cupric carbonate for 8 weeks. The copper content is 6 mg/kg in an adequate copper diet (DYET# 115612) and 1.5 mg/kg in a marginal copper deficient diet (DYET# 115581), respectively. Animals had free access to either deionized water or deionized water containing 10% fructose (w/v) as the only drink during the 8-week experiment. The animals were single housed in stainless steel cages rinsed with EDTA in a temperature and humidity-controlled room with a 12:12-h light–dark cycle. Data are expressed as means ± SD (*n* = 7–8) and analyzed by two-way ANOVA testing factors of copper (Cu), fructose (F), and interactions (Cu × F), followed by Tukey’s multiple comparison test. Statistical significance was set to *p* ≤ 0.05. *P* values are displayed for the factors Cu, F, and Cu × F. NS, *p* > 0.05. * versus CuA; ^#^ versus CuAF; ^$^ versus CuM

*CuA* adequate copper diet, *CuM* marginal copper deficient diet, *CuAF* adequate copper diet +10% fructose drinking, *CuMF* marginal copper deficient diet +10% fructose drinking, *TG* triglyceride, *NEFA* nonesterified fatty acids

**Table 2:** Effects of dietary fructose and marginal copper deficiency on metabolic phenotypes in female rats

Variable	CuA (*n* = 7)	CuAF (*n* = 8)	CuM (*n* = 7)	CuMF (*n* = 8)	*P* value of factors (two-way ANOVA)
Body weight (BW, g)	235.4 ± 13.7	220.5 ± 14	217.7 ± 17.4	220.0 ± 18.6	NS
BW gain (g)	181.2 ± 14.1	166.1 ± 13.1	163.1 ± 18.1	167.2 ± 18.1	NS
Liver weight (LW, g)	7.5 ± 0.55	7.66 ± 0.95	6.86 ± 0.77	6.92 ± 0.81	Cu, *p* = 0.0256
LW/BW (%)	3.184 ± 0.114	3.469 ± 0.26*	3.144 ± 0.133^#^	3.114 ± 0.167^#^	Cu, *p* = 0.0061 Cu × F, *p* = 0.025
White adipose weight (WAT, g)	2.961 ± 0.944	3.354 ± 0.792	2.256 ± 0.504	3.523 ± 1.309	F, *p* = 0.0239
WAT/BW (%)	1.251 ± 0.364	1.512 ± 0.285	1.032 ± 0.195	1.571 ± 0.532	F, *p* = 0.0067
Energy efficiency ratio (EER, %)	6.35 ± 0.50	5.22 ± 0.41*	6.07 ± 0.67^#^	5.36 ± 0.58*	F, *p* < 0.0001
Cecum weight (g)	2.233 ± 0.333	1.887 ± 0.489	2.107 ± 0.637	1.997 ± 0.276	NS
Food consumption (g/rat/day)	13.55 ± 1.47	10.17 ± 0.84*	12.77 ± 1.12^#^	10.39 ± 1.06*^$^	F, *p* < 0.0001
Water intake (ml/rat/day)	23.05 ± 3.92	45.91 ± 14.32*	24.46 ± 4.31^#^	41.0 ± 12.18*^$^	F, *p* < 0.0001
Energy intake (Kcal/rat/day)	50.96 ± 5.54	56.87 ± 6.89	48.01 ± 4.20^#^	55.74 ± 6.41	F, *p* = 0.0031
Plasma TG (mg/dL)	26.15 ± 4.74	39.71 ± 11.84	19.29 ± 6.14^#^	38.7 ± 13.43^$^	F, *p* = 0.0001
Plasma Cholesterol (mg/dL)	28.47 ± 23.6	36.91 ± 18.28	29.78 ± 11.89	43.76 ± 12.97	NS
Plasma NEFA (μM)	202.4 ± 33.9	289.6 ± 73.6	232.4 ± 72	267 ± 77.7	F, *p* = 0.0205
Plasma glucose (mg/dL)	111.5 ± 3.8	142.7 ± 28.1*	110 ± 8.3^#^	140.5 ± 21.8*^$^	F, *p* = 0.0001

Male and female weanling Sprague-Dawley rats from the Harlan Laboratories (Indianapolis, IN) were fed (ad lib) a modified AIN-93G purified rodent diet with defined copper content in the form of cupric carbonate for 8 weeks. The copper content is 6 mg/kg in an adequate copper diet (DYET# 115612) and 1.5 mg/kg in a marginal copper deficient diet (DYET# 115581), respectively. Animals had free access to either deionized water or deionized water containing 10% fructose (w/v) as the only drink during the 8-week experiment. The animals were single housed in stainless steel cages rinsed with EDTA in a temperature and humidity-controlled room with a 12:12-h light–dark cycle. Data are expressed as means ± SD (*n* = 7–8) and analyzed by two-way ANOVA testing factors of copper (Cu), fructose (F), and interactions (Cu × F), followed by Tukey’s multiple comparison test. Statistical significance was set to *p* ≤ 0.05. *P* values are displayed for the factors Cu, F, and Cu × F. NS, *p* > 0.05. * versus CuA; ^#^ versus CuAF; ^$^ versus CuM

*CuA* adequate copper diet, *CuM* marginal copper deficient diet, *CuAF* adequate copper diet +10% fructose drinking, *CuMF* marginal copper deficient diet +10% fructose drinking, *TG* triglyceride, *NEFA* nonesterified fatty acids

### Hepatic manifestations in response to dietary copper-fructose interaction in male and female rats

**Fig. 2:**
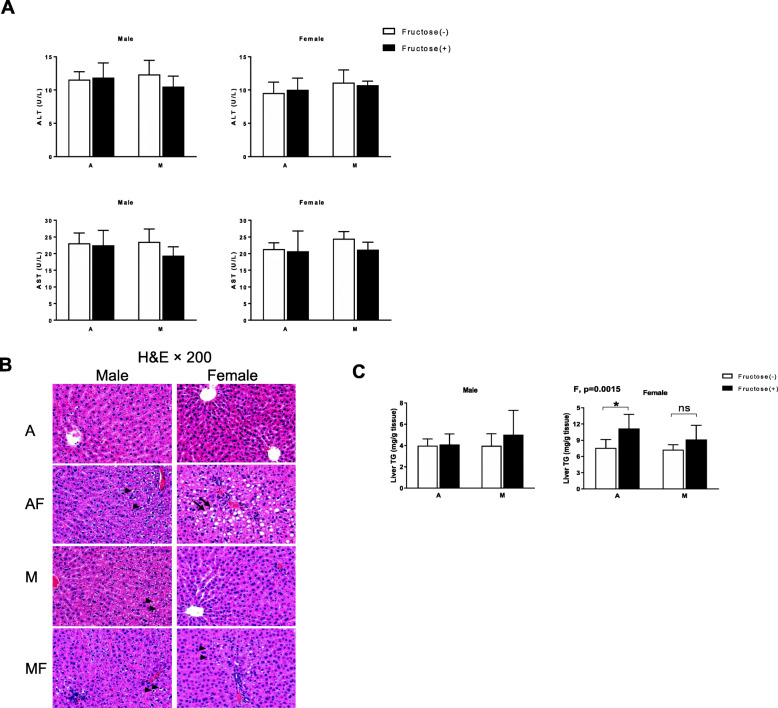
Effects of dietary copper-fructose interaction on plasma ALT, AST, liver histology, and fat accumulation. **a** Plasma ALT and AST. **b** Representative photos of liver histology using H&E staining. **c** Hepatic triglyceride. CuAF female rats had macrosteatosis (arrows) around the portal area. Microsteatosis (arrowheads) was observed in female CuMF rats as well as in some male rats as indicated. Data represent means ± SD (*n* = 7–8). Statistical significance was set at *p* ≤ 0.05. *P* values displayed are for the factors copper (Cu), fructose (F), and interaction (Cu × F) using two-way ANOVA followed with Tukey’s multiple comparisons test. *A*, adequate copper diet; *AF*, adequate copper diet +10% fructose (w/v) in the drinking water; *M*, marginal copper diet; *MF*, marginal copper diet +10% fructose (w/v) in the drinking water

Neither male nor female rats showed obvious liver injury in terms of plasma ALT and AST after being exposed to CuA or CuM diets with or without 10% fructose (w/v) for 8 weeks (Fig. 2a). Three of eight female rats fed with CuA plus fructose (CuAF) developed mild steatosis, characterized with macrosteatosis around the portal area. Only very mild microsteatosis could be visualized in either CuMF female rats or male rats fed with marginal copper diet and/or fructose (Fig. 2b). Consistently, hepatic triglyceride was significantly elevated in CuAF female rats compared to control rats (Fig. 2c). Compared to our previous study with AIN-76 diet (containing 49% sucrose) and 30% fructose (w/v) in the drinking water [21], the extent of hepatic steatosis is mild and no apparent liver injury was detected. Despite there being only mild steatosis induced under the current conditions, sex differences still were detected, with female CuAF rats showing hepatic steatosis.

### Distinct alterations of fecal gut microbiota in response to dietary copper and fructose between male and female rats as analyzed by 16S rRNA sequencing

**Fig. 3:**
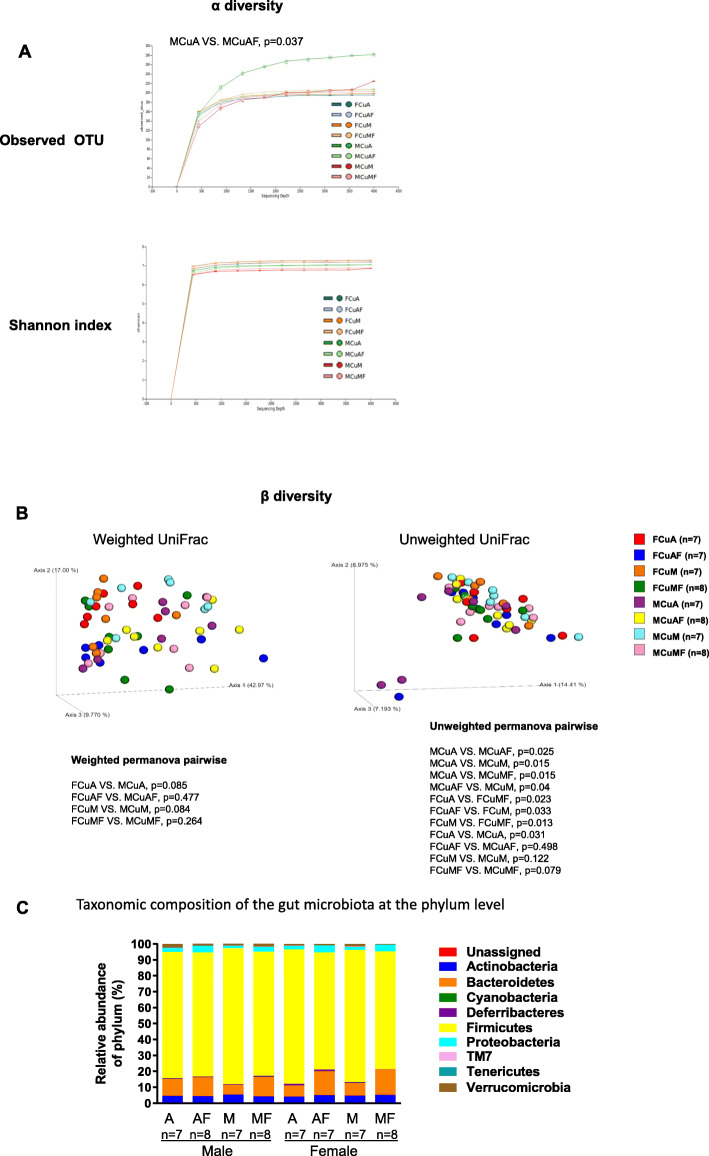
Effects of dietary copper and fructose on gut bacterial diversity and abundance. **a** Alpha-diversity: alpha rarefaction curves with each treatment using observed OTU measure and Shannon index. **b** Beta-diversity: weighted and unweighted UniFrac. **c** Taxonomic composition (percentage) of the gut microbiota at the phylum level. *Cu*, copper; *A*, adequate copper diet; *AF*, adequate copper diet +10% fructose (w/v) in the drinking water; *M*, marginal copper diet; *MF*, marginal copper diet +10% fructose (w/v) in the drinking water. M (first letter in the group name), male; F (first letter in the group name), female

To examine whether copper-fructose interaction alters the gut microbiome in a sex-specific manner, we performed 16S rRNA sequencing of fecal stool DNA. In male rats, either fructose or CuM resulted in a trend of decrease in alpha-diversity in terms of the observed OTU. However, only the difference between CuA and CuAF reached statistical significance (CuA versus CuAF, *p* = 0.037), suggesting fructose feeding led to reduced species richness in male rats [56]. There were no significant differences between groups of female rats in terms of observed OTU, suggesting neither fructose nor CuM alters the species richness of the gut microbiota in female rats. There was no significant difference between groups of both male and female rats in terms of Shannon index (Fig. 3a, supplementary Table 1). Beta-diversity was evaluated by UniFrac analysis [46]. Unweighted UniFrac is a qualitative β-diversity measure, which detects the difference in the presence or absence of lineages of bacteria in different communities [57]. Unweighted UniFrac analysis demonstrated that the mean distance between groups CuA and CuAF, CuA and CuM, and CuA and CuMF were significantly different in male rats (*p* < 0.05) (Fig. 3b, right panel, supplementary Table 2). In female rats, unweighted UniFrac analysis showed significant differences were between groups CuM and CuMF, and CuA and CuMF (*p* < 0.05) (Fig. 3b, right panel, supplementary Table 2). The weighted UniFrac measure was used for detecting differences in abundance [57], and no significant differences were detected between the four treatment groups in male or female rats (Fig. 3b, left panel, supplementary Table 2). These results suggested that either dietary fructose (CuAF) or copper (CuM) or the combined effects (CuMF) alter bacterial communities in male rats. However, bacterial communities were altered by dietary copper (CuM) or copper plus fructose (CuMF) in female rats. Moreover, the baseline bacterial communities (CuA) were significantly different between male and female rats.

**Fig. 4:**
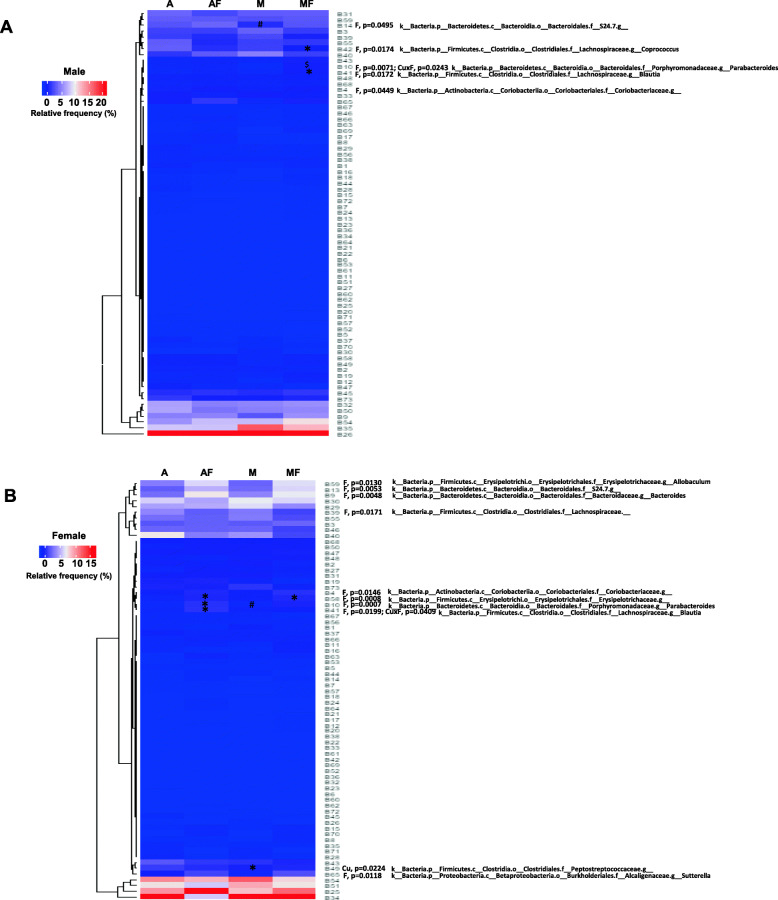
Relative abundance of gut microbiota at the genus level. Heatmap showing the abundance of 73 fecal gut microbes in **a** Male rats and **b** Female rats. Data represent means ± SD (*n* = 7–8). Statistical significance was set at *p* ≤ 0.05. *P* values displayed are for the factors copper (Cu), fructose (F), and interaction (Cu × F) by two-way ANOVA with Tukey’s multiple comparisons test. * versus CuA; # versus CuAF; $ versus CuM. *A*, adequate copper diet; *AF*, adequate copper diet +10% fructose (w/v) in the drinking water; *M*, marginal copper diet; *MF*, marginal copper diet +10% fructose (w/v) in the drinking water

At the phylum level, fructose feeding led to a remarkable increase in the abundance of Bacteroidetes and Proteobacteria and a decrease in Firmicutes independent of dietary copper content. In male rats, only the abundance of Bacteroidetes and Proteobacteria was altered by dietary fructose, and the effect was less pronounced compared to female rats (Fig. 3c, supplementary Tables 3 and 4). In agreement with this, more families and genera under the phyla Bacteroidetes, Firmicutes, and Proteobacteria were altered in female rats compared to male rats. For example, Bacteroidaceae, *Bacteroides*, Lachnospiraceae, Erysipelotrichaceae, *Allobaculum*, Alcaligenaceae, and *Sutterella* were markedly shifted in female rats, but not in male rats. Even among the commonly changed taxa, such as Porphyromonadaceae, *Parabacteroides*, and *Blautia*, the factors leading to such changes are different between males and females, as shown by two-way ANOVA (supplementary Tables 3, 4, 5, 6 and Fig. 4). In addition to the sex differences in response to dietary fructose and marginal copper, the composition of gut microbiota is also different between male and female rats when exposed to adequate copper diet, which was considered as a normal control. A higher abundance of Firmicutes and a lower abundance of Bacteroidetes were observed in female rats than in male rats, leading to a higher Firmicutes/Bacteroidetes ratio in females rats (12.06 versus 7.47, female versus male), which was considered an obese phenotype contributing to increased capacity of energy harvesting from diet [58]. Sex differences also exist in the abundance of Lactobacillaceae and *Lactobacillus* (9.39 versus 20.72, female versus male), Clostridiaceae (15.99 versus 8.69, female versus male), Ruminococcaceae (20.9 versus 17.85, female versus male), and Lachnospiraceae (17.25 versus 11.86, female versus male).

Collectively, female rats exhibit more pronounced alterations of gut microbiota, and fructose plays a dominant role.

### LEfSe identified microbiota signature associated with dietary copper and fructose

**Fig. 5:**
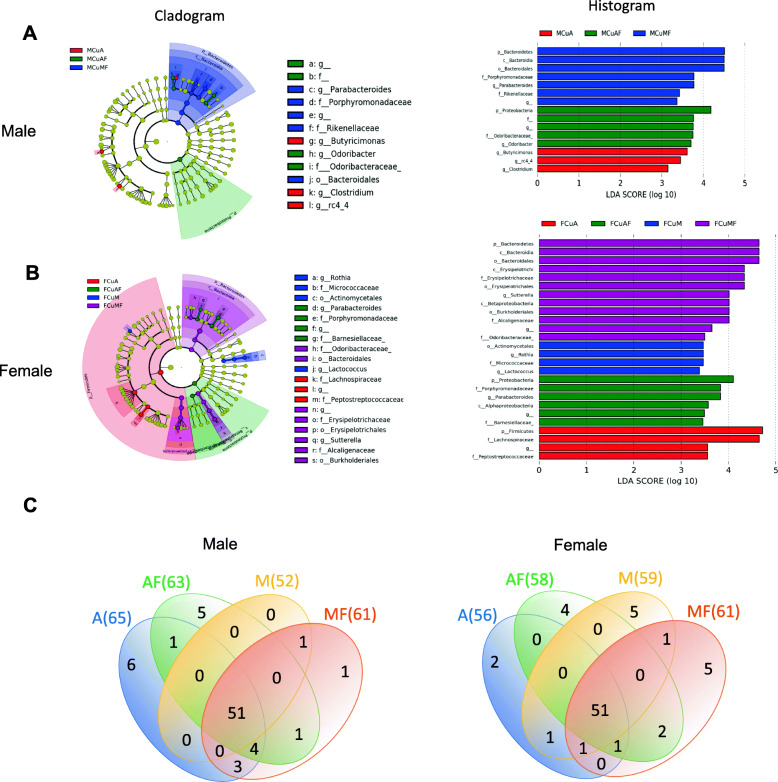
Linear discriminant analysis (LDA) effect size (LEfSe) analysis identifies differentially abundant taxa induced by dietary copper and fructose. Cladogram and histogram with LDA score ≥ 2 showing the features with differential abundance of taxa between groups in **a** male rats and **b** female rats (Wilcoxon rank-sum test). **c** Venn diagram. Each circle’s diameter in the cladogram is proportional to the taxon’s abundance. From the outer circle to the inner circle, the circles represent phyla, class, order, family, and genus. Differentially abundant taxa in specific groups were represented in different colors with the exception that yellow represents non-significant in the cladogram. *M*, male; *F*, female; *Cu*, copper; *A*, adequate copper diet; *AF*, adequate copper diet +10% fructose (w/v) in the drinking water; *M*, marginal copper diet; *MF*, marginal copper diet +10% fructose (w/v) in the drinking water

To further identify more specific taxa changes in gut microbiome by dietary copper and fructose, LEfSe analysis was performed using 16S rRNA metagenomic data [47]. Fifteen and 26 differentially abundant taxa were identified with LDA score higher than 2 in male and female rats, respectively (Fig. 5a and b). The Proteobacteria and Bacteroidetes were enriched in the CuAF and CuMF group, respectively, in both male and female rats. No specific taxa were identified to be enriched in CuM male rats. The highest number of abundant taxa was in the CuMF group (7 of 15 in male and 12 of 26 in female). Sex differences in abundance also existed in CuA rats, which were considered as normal controls. Female CuA rats were characterized by enriched Firmicutes, particularly, Lachnospiraceae. Of note, while Porphyromonadaceae and *Parabacteroides* were enriched in CuMF male rats, they were also enriched in female CuAF rats, which is consistent with the mean abundance data analysis (supplementary Tables 3 and 4). Particularly, abundant beta-Proteobacteria and Erysipelotrichi in CuMF rats as well as abundant alpha-Proteobacteria in CuAF rats were identified in female rats. Thus, distinct abundant taxa were identified by LEfSe analysis between male and females. We further performed correlation analysis between liver fat content and the genera identified by LEfSe analysis in female CuAF rats. Unfortunately, the abundance levels of the genera are not correlated with the liver fat content (supplementary figure 2).

To further explore the functional changes of gut microbiome in response to dietary copper and fructose, we performed PICRUSt2 analysis. In male rats, 40 significant differences in the functional profiles were identified by PICRUSt2 analysis between groups CuA and CuM, mainly involving fatty acid biosynthesis, electron carrier biosynthesis, lipopolysaccharide biosynthesis, and vitamin B6 biosynthesis, which were enriched in CuM male rats. Twenty-three significantly enriched pathways were predicted in male CuAF rats compared to male CuA rats. In female rats, 34 significant differences in the functional profiles were identified between CuA and CuMF groups, involving branched chain amino acid biosynthesis, fermentation, nucleotide biosynthesis and degradation, folate biosynthesis, and phospholipid biosynthesis, with lower abundance in CuMF rats (supplementary Table 7). Taken together, significant functional alterations of microbiota in female rats were induced mainly by the combined effects of copper and fructose (CuMF), whereas they were induced by either copper or fructose singly in male rats.

The Venn diagram plot showed 51 shared genera by four groups in both male and female rats. There are total 65 and 56 detected genera in male and female rats, respectively. Fructose and marginal copper led to reduced genera in male rats, but an increase in female rats. Six genera were not altered by fructose or marginal copper diet in male rats, but only two were not altered in female rats (Fig. 5c), suggesting more genera abundance changes occur in female rats.

### Sex differences in fecal short-chain fatty acids in response to dietary copper-fructose interaction

**Fig. 6:**
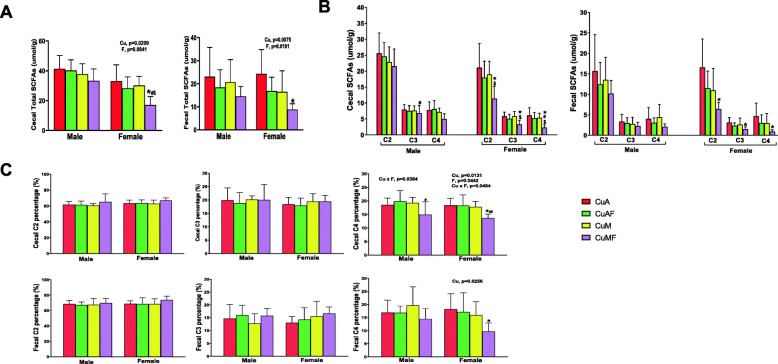
Alterations of cecal and fecal SCFA levels induced by dietary copper and fructose. **a** Total SCFA levels. **b** SCFA levels (C2–C4). **c** Percentage of total SCFAs. Data represent means ± SD (*n* = 7–8). Statistical significance was set at *p* ≤ 0.05. *P* values displayed are for the factors copper (Cu), fructose (F), and interaction (Cu × F) by two-way ANOVA with Tukey’s multiple comparisons test. * versus CuA; ^#^ versus CuAF; ^$^ versus CuM. *Cu*, copper; *A*, adequate copper diet; *AF*, adequate copper diet +10% fructose (w/v) in the drinking water; *M*, marginal copper diet; *MF*, marginal copper diet +10% fructose (w/v) in the drinking water. *C2*, acetic acid; *C3*, propionic acid; *C4*, butyric acid

To better understand the sex differences in microbial activities induced by dietary copper and fructose, we measured SCFAs by GC-MS in cecal and fecal contents. Acetate, propionate, and butyrate are the predominant SCFAs in cecal and fecal contents. Overall, the levels of total as well as individual SCFAs were higher in cecal contents than that in fecal contents in both male and female rats. While the level of total cecal SCFAs is higher in males, the level of total fecal SCFAs are comparable between male and female rats. Fructose feeding resulted in a decrease of total SCFAs in both cecal and fecal contents in CuA- and CuM-fed rats; however, a significant decrease was found in female CuMF rats. A similar trend of alterations in SCFAs, but to a lesser extent, was observed in male rats, as shown in Fig. 6a. Consistently, acetate, propionate, and butyrate were all markedly decreased in female CuMF rats (Fig. 6b). In addition, decreased total SCFAs was associated with the relatively increased proportion of acetate and decreased proportion of butyrate in both cecal (acetate to propionate to butyrate = 63.3:18.4:18.4 versus 66.9:19.5:13.6; CuA versus CuMF) and fecal stool (68.7:13.1:18.2 versus 73.7:16.6:9.7; CuA versus CuMF) of female CuMF rats. This effect was less prominent in male rats (Fig. 6c). Collectively, a substantial decrease of SCFAs was seen in female rats and profoundly so in the CuMF group. Two-way ANOVA showed that the alteration in SCFAs was most likely due to the additive effect of copper and fructose in female rats, but the decrease in SCFAs in male rats was only attributable to copper.

## Discussion

Copper-fructose interaction-induced metabolic effects exhibit sex dimorphism [23, 25]. Sex-specific alterations of gut microbiota in response to a specific diet have been demonstrated in a variety of studies [59–61]. Given that the gut microbiota play a causal role in driving the development of metabolic diseases, we aimed to determine whether sex-specific alterations of the gut microbiota are linked to hepatic steatosis. Our data showed that sex differences do exist in the gut microbiota, gut microbiota metabolites such as SCFAs, and hepatic steatosis following dietary copper and fructose exposure. Female rats exhibited more pronounced alterations in the abundance of various taxa than that did male rats at multiple taxa levels, including phylum, family, and genus. The number of distinct abundant taxa identified by LEfSe was also higher in female rats than in male rats. In addition, SCFAs were decreased to a greater extent in female rats compared to male rats, particularly in the CuMF group. Moreover, female rats with an adequate copper diet developed mild, but apparent steatosis after 8 weeks of added fructose feeding (CuAF), but female CuMF rats, which showed the most significantly altered gut microbial activity, did not. Therefore, the altered gut microbial activity does not correlate with the hepatic fat accumulation.

SCFAs are the end products of microbial fermentation of indigestible fiber, and they play a critical role in energy homeostasis and metabolism [62]. In our study, we found significantly decreased SCFAs, particularly butyrate, concomitant with the reduced butyrate producers, Lachnospiraceae and Ruminococcaceae [63], in CuMF female rats, implying that the most significantly altered gut microbial activities were in this group. We found mild hepatic steatosis in CuAF female rats; thus, it is unlikely that this hepatic steatosis is attributable to the metabolic effects of gut microbiota. Accelerated de novo lipogenesis (DNL) is known to contribute to fructose-induced hepatic steatosis [64, 65]. However, the underlying mechanisms are unclear. A recent study demonstrated a two-point mechanism leading to fructose-induced hepatic steatosis. One part is gut bacteria-derived acetate which serves as a substrate for acetyl-CoA synthesis via acyl-CoA synthetase short-chain family member 2 (ACSS2) in the liver. Second, fructose metabolism in hepatocytes activates a signal leading to lipogenic gene expression [66]. Interestingly, the most significantly changed SCFAs occurred in CuMF rats. We also observed this effect in our previous study when rats were exposed to a high-fructose diet via 30% fructose (w/v) in the drinking water and sucrose-enriched diet (AIN-76) [21]. This finding suggests that hepatic steatosis may be related to the amount of fructose intake. In support of this, a recent study demonstrated that dietary fructose is primarily metabolized in the small intestine and only excess fructose intake spills over to the colon microbiota and the liver [67]. Previous studies showed that either inhibition of fructose metabolism in the liver [68] or elimination of gut microbiota by antibiotics [69] protected against fructose-induced hepatic steatosis, indicating that fructose metabolism in both the liver and gut microbiota is required to facilitate the development of steatosis. When a large amount of fructose intake saturates the capacity of the small intestine metabolism, presumably excess fructose will proceed to the colon, the gut microbiota, and the liver. However, the priority of excess fructose to be distributed and metabolized in colon microbiota or the liver or other tissues is unclear when a modest amount of fructose was ingested. It has been shown that dietary copper-fructose interaction exacerbates copper deficiency-induced metabolic syndrome, likely due to impaired intestinal copper absorption because of excess fructose ingestion [21, 70]. Whether the extent of interaction relates to the relative amounts of copper and/or fructose, and subsequent metabolic effects, remains largely unknown and warrants further study.

Despite significantly changed gut microbiota and decreased SCFAs in CuMF rats, only a few of the female rats in the CuAF group developed modest steatosis, suggesting decreased SCFAs and the altered gut microbial activities were not sufficient to lead to hepatic steatosis in female CuMF rats. Of note, Porphyromonadaceae and *Parabacteroides* are two of the microbiota signatures associated with the CuAF diet in female rats, although with relatively low abundance (1.52%), which is different from male rats identified by LEfSe. Whether increased abundance of Porphyromonadaceae and *Parabacteroides* plays a causal role in fructose-induced hepatic steatosis needs to be examined.

Sex differences in fructose-induced metabolic effects are mixed [24, 71, 72]. In contrast to previous studies on copper-fructose interactions [23, 25, 26], our results showed that female rats are relatively sensitive to fructose-induced hepatic steatosis. The discrepancy may be attributed to several factors. First is the dose of copper and fructose. A lower dose of copper (0.6 ppm) and a higher dose of fructose (30–62%) were used in Field’s as well as in Morrell’s studies [23, 26]. It appeared that males are more sensitive to the deleterious effects of copper deficiency. In our study, marginal copper diet (1.5 ppm) and 10% fructose (w/v) in the drinking water were used, presumably leading to less-pronounced copper-fructose interactions and metabolic effects than previous studies [23, 26]. Second, the activities of fructose-metabolizing enzymes and intermediate metabolites differed by sex and copper level [73]. In fact, the activities of liver enzymes involved in lipogenesis were affected not only by the type of carbohydrate but also by the quantity [74]. Lastly, differences in facilities, diet components, and species as well as experimental durations may all contribute to discrepancy [25, 75, 76].

In support of our results, a previous study demonstrated that weanling female rats exhibit a higher rate of acetate incorporation into lipids in the liver compared to male rats [77], suggesting a higher lipogenic capacity in female rats. However, there is a different species driving the lipogenic enzyme activity in response to carbohydrate [74]. In human studies, the fructose-induced increase in hepatic DNL and decrease in fatty acid oxidation were more pronounced in men and premenopausal women than in postmenopausal women [28, 65, 78, 79]. Sex hormones are known factors regulating sex dimorphism of fructose-related metabolic effects [7]. However, the molecular underpinnings remain elusive. Recent studies showed that GLUT8 mediates distinct metabolic effects between males and females in response to dietary fructose [29, 30, 80]. GLUT8 is a dual-specificity glucose and fructose transporter, which was found to be abundantly expressed in both murine and human liver and intestine [30, 80, 81]. Interestingly, while GLUT8 mutation does not alter intestinal fructose absorption in male mice [29], it enhances intestinal fructose absorption in female mice, which was associated exacerbated hypertension, hyperinsulinemia, and hyperlipidemia in those animals when they were fed with high-fructose diet [30]. Conversely, GLUT8-deficient male mice are protected from high-fructose diet-induced dyslipidemia, glucose intolerance, and hypertension [29]. These studies revealed an important molecular mechanism underlying the tissue-specific and sex-specific divergence in response to fructose.

A potential limitation of the current study is the one time analysis of gut microbiota and hepatic steatosis. Although female rats displayed earlier development of steatosis, it is difficult to predict the ultimate severity of steatosis and disease progression. Since male rats exhibit decreased diversity of gut microbiome, and given that the microbial gene richness is associated with inflammation, insulin resistance, and dyslipidemia [82, 83], it is plausible that male rats develop steatosis with a prolonged duration on the experimental regime. Thus, long-term and multiple time points evaluation will provide more accurate profiles of disease progression in the context of sex difference. However, sex differences observed in animal studies are under strictly defined experimental conditions. Therefore, a caveat must be noted when extrapolating animal data to human, as humans have much more complex genetic and environmental factors than experimental animals.

## Perspectives and significance

In summary, our current study provides evidence of sex-specific alterations in gut microbial abundance, activities, and hepatic steatosis in response to dietary copper-fructose interaction in a rat model. However, the correlation of sex differences in hepatic steatosis and alterations of gut microbial activities was not established in the current experimental condition. Future studies deciphering the molecular mechanisms as well as tissue-specific effects would help us better understand sex-specific responses to dietary copper-fructose interactions.

## Conclusions

Our data demonstrated sex differences in the alterations of gut microbial abundance, activities, and hepatic steatosis in response to dietary copper-fructose interaction in rats. The correlation between sex differences in metabolic phenotypes and alterations of gut microbial activities remains elusive.

## Supplementary Information

**Additional file 1: Supplementary Table 1.** The Kruskal-Wallis test results between treatment groups (α-diversity).

**Additional file 2: Supplementary Table 2.** The permutation tests of the mean distance matrix (β-diversity).

**Additional file 3: Supplementary Table 3.** Mean abundance of gut microbiome taxa in male rats. **Supplementary Table 4.** Mean abundance of gut microbiome taxa in female rats. Numbers listed under study groups are percentages. Data are expressed as means ± SD (*n* = 7–8) and analyzed by two-way ANOVA testing factors of copper (Cu), fructose (F), and interactions (Cu × F), followed by Tukey’s multiple comparison test. Statistical significance was set to *p* ≤ 0.05. *P* values are displayed for the factors Cu, F, and Cu × F. NS, *p* > 0.05. CuA, adequate copper diet; CuM, marginal copper diet; CuAF, adequate copper diet +10% fructose drinking; CuMF, marginal copper deficient diet + 10% fructose drinking. * versus CuA; ^#^ versus CuAF; ^$^ versus CuM. p_Phyla, f_Families, and s_species with average abundance greater than 1% in any of the groups are listed. Unknown, 16S rRNA sequence distinct from any known genera in this family/species.

**Additional file 4: Supplementary Table 5.** Full bacteria name listed in Fig. 4a in male rats. **Supplementary Table 6.** Full bacteria name listed in Fig. 4b in female rats.

**Additional file 5: Supplementary Table 7.** PICRUSt2 analysis results.

**Additional file 6: Supplementary Table 8.** ASV table.

**Additional file 7: Supplementary Figure 1.** Schematic diagram of QIIME 2 workflow. **Supplementary Figure 2.** Correlation of liver triglyceride with signature gut bacteria in CuAF rats. Correlation of liver triglyceride with signature gut bacteria in CuAF rats. Data represent means ± SD (*n* = 7). Statistical significance was set at *p* ≤ 0.05. *P* values displayed are for Spearman correlation test.

## Data Availability

The 16S rRNA raw sequence reads are available in the National Center for Biotechnology Information (NCBI) Sequence Read Archive (SRA) with BioProject accession: PRJNA641690; BioSample accession: SAMN15358594 (https://www.ncbi.nlm.nih.gov/sra).

## Abbreviations

NAFLD: Nonalcoholic fatty liver disease
SD rat: Sprague-Dawley rat
CuA: Adequate copper diet
CuM: Marginal copper diet
F: Fructose
SCFAs: Short-chain fatty acids
LDA: Linear discriminant analysis
LEfSe: Linear discriminant analysis effect size
NASH: Nonalcoholic steatohepatitis
OVX: Ovariectomized
HFFD: High-fat high-fructose diet
ALT: Alanine aminotransferase
AST: Aspartate aminotransferase
H&E: Hematoxylin and eosin
16S rRNA: 16S ribosomal RNA
OTUs: Operational taxonomy units
ASVs: Amplicon sequence variants
PCoA: Principal coordinate analysis
WAT: White adipose tissue
EER: Energy efficiency ratio
DNL: De novo lipogenesis
ACSS2: Aacyl-CoA synthetase short-chain family member 2
C2: Acetic acid
C3: Propionic acid
C4: Butyric acid
