# Supplementary figures and images for: Analysis of sex differences in dietary copper-fructose interaction-induced alterations of gut microbial activity in relation to hepatic steatosis
Source: Biol Sex Differ. 2021 Jan 6;12:3. doi: 10.1186/s13293-020-00346-z (PMC7789350; doi:10.1186/s13293-020-00346-z)

## Slide 1
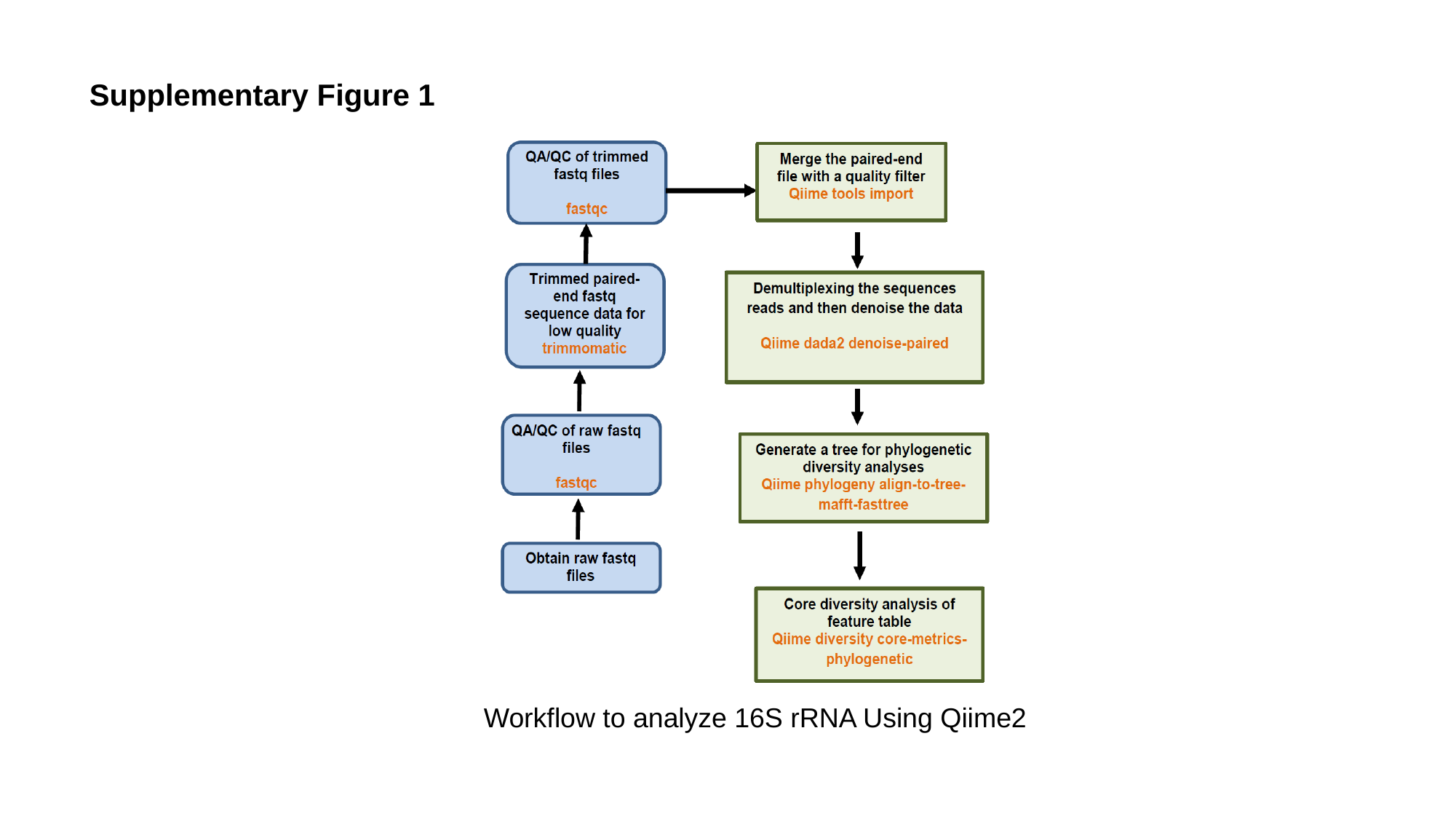

Supplementary Figure 1
Workflow to analyze 16S rRNA Using Qiime2

Supplement: Supplementary file 7 — Additional file 7: Supplementary Figure 1. Schematic diagram of QIIME 2 workflow. Supplementary Figure 2. Correlation of liver triglyceride with signature gut bacteria in CuAF rats. Correlation of liver triglyceride with signature gut bacteria in CuAF rats. Data represent means ± SD (n = 7). Statistical significance was set at p ≤ 0.05. P values displayed are for Spearman correlation test. [file 13293_2020_346_MOESM7_ESM.zip › Fruc&CuM-sex difference supplementary figure-1.pptx]

## Slide 1
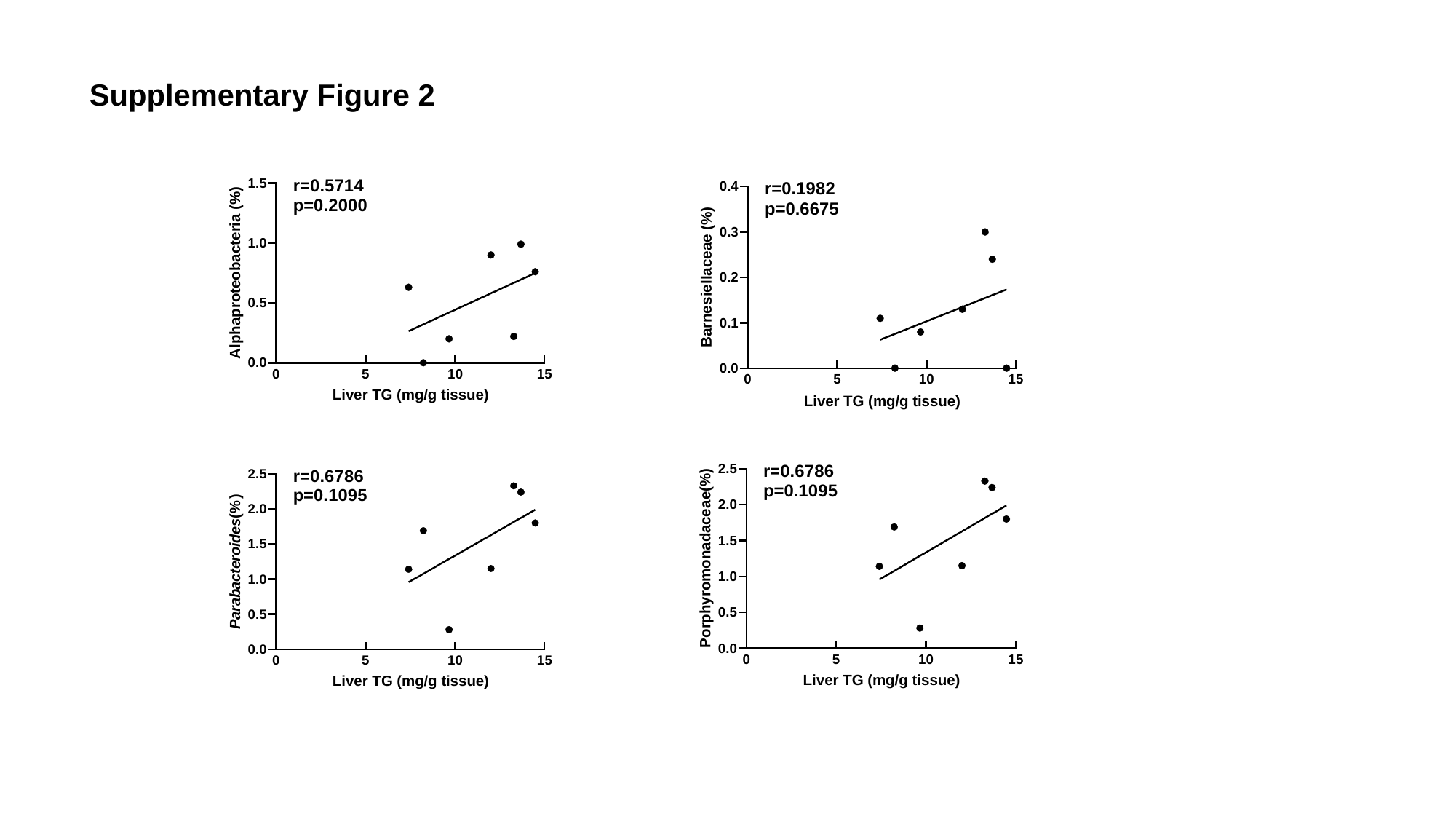

Supplementary Figure 2

Supplement: Supplementary file 7 — Additional file 7: Supplementary Figure 1. Schematic diagram of QIIME 2 workflow. Supplementary Figure 2. Correlation of liver triglyceride with signature gut bacteria in CuAF rats. Correlation of liver triglyceride with signature gut bacteria in CuAF rats. Data represent means ± SD (n = 7). Statistical significance was set at p ≤ 0.05. P values displayed are for Spearman correlation test. [file 13293_2020_346_MOESM7_ESM.zip › Fruc&CuM-sex difference supplementary figure-2.pptx]
